# Gender Differences in On-Site and Online Gambling Among Finnish Adolescents: Associations with School-, Family-, and Peer-Related Factors and Other Risk Behaviors

**DOI:** 10.3390/ijerph23060753

**Published:** 2026-06-03

**Authors:** Sari Castrén, Johanna Järvinen-Tassopoulos, Kirsimarja Raitasalo

**Affiliations:** 1 Department of Health Care and Social Welfare, Finnish Institute for Health and Welfare, 00271 Helsinki, Finland; johanna.jarvinen-tassopoulos@thl.fi (J.J.-T.); kirsimarja.raitasalo@thl.fi (K.R.); 2 Department of Psychology and Speech-Language Pathology, University of Turku, 20014 Turku, Finland; 3 Department of Public Health, Faculty of Medicine, University of Helsinki, 00014 Helsinki, Finland; 4 Centre for Research on Addiction, Control, and Governance, Faculty of Social Sciences, University of Helsinki, 00014 Helsinki, Finland; 5 Department of Social Sciences, University of Eastern Finland, 80101 Joensuu, Finland

**Keywords:** adolescence, youth, gambling, risk behaviors, problem gambling

## Abstract

**Highlights:**

**Public health relevance—How does this work relate to a public health issue?**

- Adolescent gambling is linked with multiple risk behaviors, including substance use, truancy, and risky sexual behavior, indicating that it forms part of a broader pattern of youth risk-taking with potential long-term health and social consequences.
- The increasing digitalization of gambling and exposure through online environments and social media may increase adolescents’ vulnerability, making youth gambling an emerging concern for public health.

**Public health significance—Why is this work of significance to public health?**

- The study highlights the importance of social environments, such as parental monitoring, peer activities, and school engagement, in shaping adolescents’ gambling behaviors, identifying key leverage points for prevention.
- Gender differences in gambling participation and risk pathways suggest that prevention strategies may need to be tailored differently for boys and girls to effectively reduce gambling-related harms.

**Public health implications—What are the key implications or messages for practitioners, policy makers and/or researchers in public health?**

- Prevention strategies should adopt a multi-component approach that strengthens parental monitoring, supports school engagement, and addresses co-occurring risk behaviors rather than focusing on gambling alone.
- Policymakers should continue to enforce and strengthen measures preventing underage access to gambling, particularly in digital environments, while researchers should further investigate gender-specific pathways and protective factors in youth gambling.

**Abstract:**

Adolescent gambling is a growing public health concern as opportunities expand across both physical and digital environments. This study examined gender differences in on-site and online gambling among Finnish adolescents and assessed associations with school engagement, family context, peer activities, and co-occurring risk behaviors. Data were obtained from the European School Survey Project on Alcohol and Other Drugs (ESPAD), collected from Finnish adolescents aged 15–16 in 2024 (boys: *n* = 1706; girls: *n* = 1588). Associations with past-12-month gambling were analyzed using Rao–Scott’s chi-square tests, F tests, and multinomial logistic regression, examining gender interactions. Gambling was more common among boys than girls: 7% of boys had gambled on-site only, 3% online only, and 11% both during the past 12 months. Among girls, the corresponding proportions were 0–2%. Skipping school, spending leisure time with friends, risky sexual behavior, and problematic substance use were associated with increased odds of both gambling types, whereas parental control was associated with decreased odds. Problematic social media increased the odds of on-site gambling, while problematic gaming decreased the odds. Several associations with online gambling differed by gender. These findings support multi-level prevention targeting family, school, peer, and behavioral risk factors.

## 1. Introduction

Adolescence is the period of transition from childhood to adulthood defined by biological, social, psychological, and economic development. This period is a developmental stage characterized by heightened risk-taking and exploration eased by greater autonomy and the charm of new experiences [1,2,3,4]. Early engagement in gambling is highly concerning, since underage gambling poses a risk for developing gambling problems later in life [5]. Today’s adolescents not only encounter traditional on-site gambling (i.e., in Finland, in grocery and department stores, gas stations) but also increasing opportunities of online gambling forms signaling a rapid growth of legalized online gambling options [6,7].

Although gambling is legally prohibited for minors, participation among adolescents remains a concern. According to 2024 data, gambling among Finnish youth is less prevalent than the European average: 9.8% of adolescents in Finland reported on-site gambling and 7.3% online gambling, compared to 19.4% and 14.5%, respectively, across Europe [8]. National figures further show that Finnish boys are significantly more likely than girls to gamble, both on-site and online. Slot machine use in commercial spaces and engagement in online gambling, particularly on foreign offshore gambling sites, are especially common among boys. Being male is one of the most consistent predictors of higher gambling participation and problem gambling during adolescence [9,10,11,12]. Previous research has also suggested that gender differences may extend beyond gambling prevalence alone, influencing motivations for gambling, social contexts of participation, and vulnerability to gambling-related harms. In addition, studies on behavioral addictions have highlighted gender-specific psychological and behavioral mechanisms, supporting the importance of examining gender-specific patterns during adolescence [13,14]. 

Many adolescents who gamble online report using both domestic and foreign sites, indicating that platform restrictions are often circumvented [12]. Despite existing age restrictions, research has consistently shown that adolescents in Europe continue to engage in gambling activities, both on-site and online [8,15,16,17]. In general, youth are widely exposed to gambling through multiple channels, including advertising, sports content, and digital games that feature gambling-like mechanics [17,18,19]. This may reflect the broader normalization of gambling in society, where it is commonly viewed as a socially acceptable activity and frequently presented in a positive light, particularly through advertising [18,19,20].

The normalization of gambling among adolescents is often reinforced through family and social environments. Adult behavior plays a particularly important role in shaping young people’s perceptions of gambling. Adolescents frequently encounter gambling through their parents’ behavior, either through direct participation or through permissive attitudes at home. Research has shown that parental gambling and tolerance toward youth gambling can contribute to adolescents perceiving gambling as acceptable, whereas parental disapproval is associated with more critical attitudes toward gambling [20]. Previous studies have also documented that parents may actively facilitate adolescents’ participation by allowing gambling at home, purchasing lottery tickets or scratch cards as gifts, or even gambling on behalf of their children [18,21]. A recent survey in Australia further highlighted how parental behavior contributes to normalization: although most parents disapprove of underage gambling, many view it as less serious than other youth risk behaviors such as drug use or bullying, and a considerable proportion, particularly fathers, report gambling in front of their children [18].

Peer environments also play a significant role in reinforcing gambling-related norms and behaviors during adolescence. Young people often receive information, encouragement, and social cues related to risky behaviors, including gambling, through their friends as well as through social media and online platforms [1,3]. Within peer groups, gambling may function as a social or bonding activity, particularly in contexts where betting is normalized and a shared interest [20,22]. Consistent with these social influences, research has identified several demographic and contextual factors associated with adolescent gambling. These findings highlight how gender, family environments, and peer dynamics together shape adolescents’ exposure to and perceptions of gambling.

Beyond social influences, individual and psychosocial factors contribute substantially. Gambling often co-occurs with other risky behaviors, including substance use, and may be linked to problematic use of social media or gaming and risky sexual activity, suggesting a broader pattern of risk behavior clustering during adolescence [3,23,24,25,26,27,28]. This clustering of risk behaviors may also be interpreted within the broader framework of Problematic Usage of the Internet (PUI), which recognizes online gambling, gaming, and social media use as potentially interconnected online activities that may share common psychosocial and mechanisms [29]. Previous research has also suggested that gaming and gambling may share overlapping psychological and behavioral mechanisms, while gambling-like features embedded in digital games may increase adolescents’ familiarity with gambling-related reward structures [30]. School truancy, while not always classified as a risky behavior per se, is an important indicator of school disengagement and has been associated with a range of adverse outcomes [31]. When truancy becomes a recurring issue, it tends to cluster with other vulnerabilities particularly among adolescents already facing multiple risks making it a useful proxy for broader behavioral and academic challenges.

### Current Study

This study builds on existing research by examining factors associated with adolescents’ engagement in both on-site (i.e., grocery and department stores, gas stations) and online gambling. While prior studies have identified general correlates of youth gambling, less is known about whether these differ across gambling modalities, especially in the context of rapidly expanding online access. Focusing on a Nordic setting where gambling is culturally present yet age-restricted, this study explores a range of demographic, familial, peer, and behavioral factors that may influence gambling involvement.

The study examines (1) gender differences in adolescents’ participation in on-site and online gambling, (2) the associations of school engagement, family context, peer activities, and co-occurring risk behaviors with on-site and online gambling, and (3) whether these associations differ by gender.

## 2. Materials and Methods

### 2.1. Data

The analysis was based on Finnish data from the European School Survey Project on Alcohol and Other Drugs (ESPAD), comprising a representative cross-section of young people born in 2008. The data were collected in spring 2024. The definition of the target population was students, who (1) were enrolled in regular education, (2) turned sixteen during the calendar year of the survey, and (3) were present in class on the day the survey was administered. This definition included students in regular or general education, but excluded students attending special schools for those with learning disorders or severe physical disabilities, as well as students unable to respond in either of Finland’s two official languages, Finnish or Swedish.

Nationally representative samples were collected using a two-stage systematic probability-proportional-to-size sampling method, with Nomenclature of Territorial Units for Statistics 2 regions (NUTS 2) used as strata and schools as clusters. The data were collected through an online questionnaire, which students completed in a classroom setting. Participation was voluntary, and anonymity was ensured [8,32]. Both the school participation rate and the student response rate were approximately 90%. Respondents who did not belong to the target population, as well as those who had responded inconsistently, clearly exaggerated their answers, or left more than half of the questionnaire unanswered, were excluded from the data. This resulted in a final analytical sample of 3294 respondents (boys: *n* = 1706; girls: *n* = 1588).

### 2.2. Measures

#### 2.2.1. Outcomes

Past-12-month gambling was assessed with the question “How often (if ever) did you gamble for money in the last 12 months?”, with the response options: 1 = I have not gambled for money during the last 12 months; 2 = monthly or less; 3 = 2–4 times a month; 4 = 2–3 times or more a week. Those reporting any gambling were coded as 1, and the others as 0.

Past-12-month on-site/online gambling was assessed with similarly formulated questions for both: “If you have gambled for money ON-SITE (in physical places such as bars and clubs and in the Finnish context grocery and department stores, gas stations)/ONLINE in the last 12 months, which games have you played?” (Separate lines were provided for slot machines, playing cards, lotteries, betting.) The response options for each game type in both questions were 1 = I have not played these games; 2 = monthly or less; 3 = 2–4 times a month; 4 = 2–3 times or more a week. Those reporting any on-site/online gambling were coded as 1 and the others as 0, respectively.

#### 2.2.2. Independent Variables

Plans for post-compulsory education were assessed with the question “Where are you aiming to study after comprehensive school?” with the following response alternatives: 1 = general upper secondary school; 2 = vocational school/training; 3 = extra training aiming at secondary education (TUVA); 4 = double degree (vocational education where upper secondary school studies can also be performed); 5 = a year at folk high school; 6 = other; 7 = I don’t know. Those answering general upper secondary school (aiming at high school) were coded as 1 and all others as 0.

Truancy was assessed with the question: “During the last 30 days, on how many days have you missed one or more lessons because you skipped?” (1 = “none”, 2 = “1 day”, 3 = “2 days”, 4 = “3–4 days”, 5 = “5–6 days”, 6 = “7 days or more”.) Those reporting any truancy were coded as 1; those reporting “none” were coded as 0.

Parental monitoring was assessed with the question: “Do your parents know where you spend Friday nights?” (The response alternatives were “always know”, “quite often known”, “sometimes know”, “usually do not know”.) Those answering “always know” or “quite often know” were coded as 1 (“high parental monitoring”), others were encoded as 0 (“low parental monitoring”).

Risky sexual behavior was assessed with the following question: “During the last 12 months have you experienced the following (engaged in sexual intercourse without a condom)?” The response options were 1 = never; 2 = yes, while using alcohol; 3 = yes, while using drugs; and 4 = yes, but not while using alcohol/drugs. Those responding “never” were coded as 0 and the others as 1.

A sum variable for past-30-day substance use was constructed from the questions on alcohol and cannabis use (On how many occasions (if any) have you (1) had any alcoholic beverage to drink/(2) used cannabis during the past 30 days?) and tobacco and other nicotine product use (Have you ever smoked cigarettes/used e-cigarettes/used moist snuff (snus)/used nicotine pouches?). The response options for the alcohol and cannabis questions ranged from 1 (never) to 7 (40 or more times). For all these products, any use was coded as 1 and no use as 0. The responses for all these questions were summed and thus ranged from 0 to 6.

Summary indexes for problematic social media use/problematic gaming were calculated (range 0–3). These non-clinical screening tools [33] focus on a student’s perception of problems related to the time spent on social media/gaming, bad feelings in the case of restricted access, and parental concerns. The students were asked two separate questions as to what extent they agreed with three statements on these behaviors (“I think I spend way too much time on social media/playing games”, “I get in a bad mood when I cannot spend time in social media/on games”, “My parents say that I spend way too much time in social media/on gaming”, with the response categories being “strongly agree”, “partly agree”, “neither agree nor disagree”, “partly disagree” and “strongly disagree”. Positive answers (“strongly agree” and “partly agree”) were summed to produce an index score. A 0–1-point index score was considered to indicate a low level of self-perceived problems, and a score of 2–3 points was considered to indicate a high level of self-perceived problems related to social media use/gaming.

Going out with friends weekly was assessed with a sum variable constructed from two questions: “How often (if at all) do you (1) go out with friends in the evening (to a cafe, restaurant, movies, etc.) and (2) go around with friends to shopping centres, streets, parks, etc., just for fun”, with five response alternatives for each ranging from never to almost every day. The responses to these questions were summed up and divided by two.

A proxy for socioeconomic situation (SES) was constructed from the question “How well off is your family compared to other families in your country?” with the response options 1 = very much better off; 2 = much better off; 3 = better off; 4 = about the same; 5 = less well off; 6 = much less well off; 7 = very much less well off. Based on their responses, the students were divided into three categories (better off/same/worse off).

The associations of original continuous independent variables with each other are presented in a correlation matrix (Appendix A).

### 2.3. Statistical Analysis

First, cross-tabulations with Rao–Scott’s chi-square tests for the dichotomous variables and means with standard errors and F tests for the continuous variables were applied to study the associations of the background factors with gambling in the past 12 months separately for boys and girls.

Second, multinominal logistic regression models were fitted separately for gambling on-site/online in the past 12 months, adjusting for all independent variables. Odds ratios with 95% confidence intervals were then derived from the full model as marginal estimates. Interactions with gender were tested to see and study the moderating effect of gender, that is, whether the effects were different for boys and girls. *p* values for the interactions were obtained from the full model.

The sample design was considered in all analyses by adjusting the student-level standard errors for clustering effects. This was carried out using the survey procedures in SAS EG 8.5 [34].

## 3. Results

Table 1 shows that among those who have participated in gambling (gamblers) during the past 12 months, fewer are aiming at high school and more are skipping school than among those who have not participated in gambling (non-gamblers). Parents are better aware of their children’s whereabouts among non-gamblers than among gamblers. Risky sexual behaviors, as well as problematic substance use, problematic social media use, and problematic gaming are more common among gamblers than non-gamblers. Also, leisure time spent hanging around with friends is more common among gamblers. The same results apply to both boys and girls, although there are no differences regarding problematic social media use and problematic gaming.

Table 2 shows that both on-site and online gambling are clearly more common among boys than among girls. As the numbers of girls who have participated in on-site or online gambling only are small, further analyses were conducted only for those who had gambled on-site (only or also online) and for those who had gambled online (only or also on-site).

Table 3a shows that when adjusted for all study variables, boys have almost 10-fold odds of on-site gambling compared to girls. However, no significant interactions with gender and other independent variables were observed, meaning that the effects of the studied variables on on-site gambling are similar for boys and girls. Aiming at high school is not associated with on-site gambling, but skipping school increases the odds (OR = 1.42). Both lower and higher family SES are associated with increased odds of on-site gambling compared with those with the same SES (OR = 1.81 and OR = 1.65, respectively). Furthermore, parental monitoring (awareness of Friday nights) is linked with decreased odds of on-site gambling (OR = 0.64), but hanging around with friends in leisure time is associated with increased odds (OR = 1.39). Risky sexual behavior (OR = 1.70) as well as substance use (OR = 1.30) are also associated with increased risk of on-site gambling. Finally, problematic social media use is associated with increased odds of on-site gambling (OR = 1.18), whereas the reverse is true for problematic gaming (OR = 0.83).

**Table 3:** (**a**). Factors associated with gambling on-site during the past 12 months among Finnish 15–16-year-old adolescents in 2024, unadjusted (OR) and adjusted (AOR) odds ratios with 95% confidence levels (CL). (**b**). Factors associated with gambling online during the past 12 months among Finnish 15–16-year-old adolescents in 2024, unadjusted (OR) and adjusted (AOR) odds ratios with 95% confidence levels (CL).

(a)				
	Model 1		Model 2 (Model 1 + All Independent Variables)	
Effect	OR	95% CL	AOR	95% CL
Gender (ref = girl)	7.58	5.30–10.83	9.65	6.30–14.76
Aiming at high school (ref = no)	0.63	0.49–0.81	1.24	0.90–1.71
Truancy (ref = no)	1.79	1.37–2.34	1.42	1.00–2.00
SES better (ref = same)	2.39	1.86–3.07	1.81	1.33–2.46
SES worse (ref = same)	2.02	1.36–2.99	1.65	1.06–2.57
High parental monitoring (ref = no)	0.33	0.24–0.45	0.64	0.44–0.94
Hanging around with friends (ref = no)	1.85	1.60–2.14	1.39	1.19–1.63
Risky sexual behavior (ref = no)	3.15	2.27–4.38	1.70	1.16–2.48
Substance use 30 days/sum	1.55	1.44–1.66	1.30	1.18–1.43
Problematic social media use 12 months/sum	0.95	0.84–1.04	1.18	1.01–1.38
Problematic gaming 12 months/sum	1.11	0.97–1.27	0.83	0.69–0.99
(**b**)				
	**Model 1**		**Model 2 (Model 1 + All** **Independent Variables)**	
**Effect**	**OR**	**95% CL**	**AOR**	**95% CL**
Gender (ref = girl)	13.35	7.81–22.81	14.48	8.11–25.83
Aiming at high school (ref = no)	0.58	0.44–0.77	1.33	0.91–1.95
Truancy (ref = no)	1.86	1.40–2.49	1.45	1.01–2.07
SES better (ref = same)	2.53	1.89–3.38	1.89	1.34–2.68
SES worse (ref = same)	2.27	1.48–3.47	1.80	1.07–3.01
High parental monitoring (ref = no)	0.32	0.23–0.44	0.65	0.45–0.94
Hanging around with friends (ref = no)	1.73	1.45–2.05	1.23	1.02–1.47
Risky sexual behavior (ref = no)	3.50	2.50–4.88	1.80	1.19–2.70
Substance use 30 days/sum	1.62	1.50–1.76	1.41	1.26–1.58
Problematic social media use 12 months/sum	0.92	0.79–1.06	1.08	0.89–1.32
Problematic gaming 12 months/sum	1.21	1.05–1.40	0.92	0.75–1.12

Table 3b shows that when adjusted for all study variables, boys have over a 14-fold risk of online gambling compared to girls. Aiming at high school is not associated with online gambling, but truancy is associated with increased odds (OR = 1.45). Both lower and higher family SES are linked with increased odds of online gambling compared with those with the same SES (OR = 1.89 and OR = 1.80, respectively). Furthermore, parental monitoring is associated with decreased odds of online gambling (OR = 0.65), but hanging around with friends in leisure time is linked with increased odds (OR = 1.23). Also, risky sexual behavior is associated with increased risk of online gambling (OR = 1.80), as is problematic substance use (OR = 1.41). Problematic social media use and problematic gaming are not associated with online gambling.

Significant interactions between gender and school factors (aiming at high school (*p* = 0.0201) and truancy (*p* = 0.0404)) were observed, meaning that the effects of these factors on online gambling are different for boys and girls. This being the case, separate analyses for boys and girls were conducted (Table 4).

## 4. Discussion

Using a large sample of Finnish adolescents, this study examined gender differences in participation in on-site and online gambling. The results highlight the role of school engagement, family context, peer activities, and co-occurring risk behaviors in relation to gambling involvement. By also assessing whether these associations differed between girls and boys, the study offers a more detailed picture of gendered patterns of adolescent gambling. Adolescents who reported gambling in the past 12 months were more likely to engage in risky sexual behavior, substance use, and truancy, and they also reported lower levels of parental monitoring [3,23,24,25,26,27,28]. These patterns were evident among both boys and girls, suggesting that gambling is linked to broader psychosocial vulnerability irrespective of gender. Taken together, these findings indicate that adolescent gambling is not an isolated behavior, but rather part of a wider constellation of risk behaviors. In line with earlier studies, our results also suggest that gambling during adolescence tends to cluster with poor school engagement, weaker parental control, and active participation in peer-centered, unstructured leisure activities [1,3,20,22]. Interestingly, the lack of gender differences in the associations of problematic social media use and gaming to gambling suggests that while these behaviors are prevalent among youth [35,36], their association with general gambling behavior remains unclear and may vary depending on the mode of gambling.

The findings suggest that on-site and online gambling among adolescents follow partly distinct patterns. While boys were substantially more likely than girls to engage in both forms of gambling, gender differences were particularly pronounced for online gambling as found earlier [9,10,11]. Despite this large gender gap, the associations between most social and behavioral factors and on-site gambling did not differ by gender, indicating that similar mechanisms may underlie live gambling participation among boys and girls.

Several contextual factors were associated with both gambling modalities, highlighting the role of adolescents’ social environments [28,37,38]. Truancy, spending leisure time hanging around with friends, and engagement in other risk behaviors, such as substance use and risky sexual behavior, were consistently linked to increased gambling participation. These findings support previous research suggesting that adolescent gambling tends to cluster with other risk behaviors and may form part of a broader problem behavior pattern [3,23,24,25,26,27,28]. In addition, previous research has suggested that peer-group belonging and participation in gambling- and gaming-related online communities may further reinforce gambling behaviors among adolescents through social identification and online identity-bubble processes [39]. Prospective research has also shown that peer pressure, perceived peer gambling, and peer approval of gambling are associated with higher odds of gambling initiation among adolescents [40]. At the same time, parental monitoring appeared to have a potentially protective effect, as greater parental awareness of adolescents’ whereabouts was associated with lower odds of both gambling types [20,22].

The results also indicate some modality-specific patterns. Problematic social media use was associated with on-site gambling, whereas problematic gaming was negatively associated with it. For online gambling, similar associations were not found. These results suggest that different digital leisure activities may relate differently to gambling engagement.

Gender-stratified analyses showed further divergence. Among boys, the same set of predictors remained relevant for online gambling as when both genders were studied together. Among girls, however, only truancy and substance use remained significantly associated, suggesting a narrower and potentially more selective risk pathway. Significant interactions between gender and school-related factors, particularly educational aspirations and truancy, highlight that the role of school engagement in online gambling differs by gender. For boys, online gambling appears more embedded in a wider profile of school disengagement, while for girls, it may be influenced by other, unmeasured psychosocial dynamics.

Overall, these findings highlight that adolescent gambling cannot be understood as a single behavior but rather as a set of activities embedded in different social contexts and risk profiles. Although the previous literature has suggested potential overlap between gaming, online gambling, and other internet-mediated activities through shared psychosocial and behavioral mechanisms [30], the present findings did not demonstrate consistent associations between these activities across gambling modalities. This may indicate that the relationships between gaming, social media use, and gambling are more context- and modality-specific during adolescence than broader theoretical frameworks alone might suggest. Nevertheless, the broader framework of Problematic Usage of the Internet (PUI) [29] remains useful for understanding how different online activities may coexist within adolescents’ digital environments and potentially interact with other risk-related behaviors. Distinguishing between gambling modalities may therefore be important when designing prevention strategies targeting youth gambling.

### 4.1. Implications and Further Research

The findings of this study have several implications for prevention, policy, and research. Given the consistent associations between adolescent gambling and other risk behaviors, including substance use, truancy, and risky sexual activity, prevention efforts should adopt a multi-component approach. Interventions that promote school engagement, strengthen parental monitoring, and address co-occurring behavioral risks are likely to be most effective. Parents play a particularly important role in shaping adolescents’ choices. Open communication, supervision, and positive role modeling have been shown to protect against youth gambling and other risky behaviors [1,18]. Yet prior research has found that many parents underestimate or overlook their children’s gambling behaviors and view them as relatively harmless compared to other issues such as substance use [41]. This gap in awareness may contribute to gambling’s normalization in some families [20,42]. Future studies could further examine the role of parent–child attachment and emotional closeness in adolescent gambling behaviors, as secure attachment relationships may function as protective factors against risky behaviors and gambling involvement [43].

At the policy level, stronger regulation of underage access to gambling, especially online, is warranted. In Finland, the legal age limit for all gambling products was unified at 18 years between 2009 and 2011, and mandatory identification for gambling was introduced in 2022. These regulatory changes have been associated with a substantial decline in adolescents’ gambling in convenience stores, supermarkets, and kiosks, particularly in slot machines [9]. However, despite these measures, some underage youth continue to gamble, indicating ongoing challenges in fully preventing access. Czako et al. [44] argue the need to evaluate existing prevention measures, tailor interventions to populations, and strengthen the evidence base for policy and early intervention in gambling harm.

Moreover, widespread exposure to gambling marketing through digital and social media platforms may undermine the effect of preventive work and legislative measures, especially for youth who are already vulnerable [45]. As Hing et al. [22] emphasize, abstaining from gambling is shaped by multiple social and environmental influences, including peer and parental discouragement, access restrictions, and critical attitudes developed through financial literacy and media awareness. Their findings suggest that youth who do not gamble often have a combination of protective factors such as hobbies, trusted adults, and rational gambling beliefs, highlighting the importance of building strengths, not just addressing risks.

Future research should continue to explore these dynamics using longitudinal designs to better understand causal pathways between adolescent behaviors and later gambling outcomes. Qualitative studies, such as Hing et al.’s grounded theory model [22], provide valuable insights into the protective factors that deter youth from gambling and can inform tailored interventions. Further investigation is also needed to understand why problematic gaming is negatively associated with on-site gambling, and how girls’ gambling pathways may differ from those of boys. A deeper understanding of these nuances will be crucial for designing gender- and context-sensitive prevention strategies in an evolving digital gambling landscape.

The Finnish gambling system is currently undergoing a significant structural reform aimed at opening the national gambling market to competition. From July 2027 onwards, licensed operators will be able to offer online casino games (including electronic gambling machines) and sports betting to Finnish citizens aged 18 years and older. These developments are expected to increase marketing exposure and gambling availability, highlighting the importance of strong harm-prevention frameworks [6,46]. There is also evidence that a subgroup of individuals who later develop gambling problems often report initiating gambling at an underage age, suggesting heightened vulnerability among certain youth populations [47] although there are contrasting findings [48]. In this evolving regulatory environment, it will be particularly important to ensure that gambling marketing does not disproportionately target adolescents and young adults, and that effective measures remain in place to prevent underage gambling [44].

### 4.2. Limitations

Several limitations should be considered when interpreting the results of this study. First, surveys are often criticized for failing to reach the most vulnerable or hard-to-reach segments of the population—for example, youth who have dropped out of school or who are particularly susceptible to gambling and other risk behaviors. In this case, however, the response rate was high, the entire target group (15- to 16-year-olds) was still enrolled in school, and no issues related to school participation were identified [8]. As with all self-reported data, there is a risk that students may consciously or unconsciously provide inaccurate or incomplete responses. Such inaccuracies can occur in either direction; depending on social norms and contextual factors, some behaviors may be over-reported while others are under-reported. Nonetheless, a validity assessment of ESPAD data [49] indicates that only a very small minority (1–2%) of respondents fail to answer honestly to questions on substance use. There is no reason to assume that response patterns would differ for questions related to gambling. In addition, along with the already mentioned limitation related to the cross-sectional study design, the restricted availability of psychosocial variables within the ESPAD dataset limited more detailed examination of underlying psychological and social mechanisms associated with adolescent gambling behaviors. Finally, due to the cross-sectional design of the survey, no causal inferences can be drawn regarding associations between the examined factors and gambling behaviors.

## 5. Conclusions

This study contributes to a growing body of evidence showing that adolescent gambling is closely linked with a broader pattern of risk behaviors and social influences. Both on-site and online gambling were significantly more common among boys and were associated with truancy, risky sexual behavior, substance use, and peer-centered leisure activities. Parental monitoring emerged as a consistent protective factor. Problematic gaming was negatively associated with on-site gambling, although it was not related to online gambling. Importantly, gender-stratified analyses suggest that the pathways to gambling may differ between boys and girls, underscoring the need for targeted prevention and intervention efforts, and longitudinal studies exploring these pathways.

These findings highlight the importance of multi-layered prevention strategies that strengthen protective factors at the family, school, and peer levels, while addressing structural influences such as access, advertising, and the normalization of gambling. As gambling opportunities become increasingly integrated into digital environments, ongoing research and proactive public health efforts are needed to protect adolescents from gambling-related harms.

## Figures and Tables

**Table 1:** The prevalence (%) with Rao–Scott chi-square tests and means with standard errors of studied background factors according to gambling participation during the past 12 months, total and by gender.

	Total			Boys			Girls		
	Gambling 12 Months, %			Gambling 12 Months, %			Gambling 12 Months, %		
	No (*n* = 2470)	Yes (*n* = 688)	*p* (chisq)	No (*n* = 1042)	Yes (*n* = 556)	*p* (chisq)	No (*n* = 1428)	Yes (*n* = 132)	*p* (chisq)
Aiming at high school	63%	46%	<0.001	55%	44%	<0.001	70%	58%	0.008
Truancy	21%	34%	<0.001	13%	30%	<0.001	27%	50%	<0.001
SES/better	34%	48%	<0.001	39%	50%	<0.001	30%	38%	0.043
SES/same	56%	40%		53%	39%		58%	46%	
SES/worse	10%	12%		9%	11%		12%	16%	
High parental monitoring	92%	80%	<0.001	93%	81%	<0.001	91%	74%	<0.001
Risky sexual behavior	9%	17%	<0.001	6%	17%	<0.001	11%	21%	0.004
	Mean (se)	Mean (se)	Pr (F)	Mean (se)	Mean (se)	Pr (F)	Mean (se)	Mean (se)	Pr (F)
Hanging around with friends	3.27 (0.02)	3.72 (0.04)	<0.001	3.18 (0.04)	3.71 (0.04)	<0.001	3.33 (0.03)	3.75 (0.08)	<0.001
Substance use	0.65 (0.03)	1.67 (0.09)	<0.001	0.58 (0.04)	1.66 (0.10)	<0.001	0.71 (0.04)	1.73 (0.18)	<0.001
Problematic social media use	1.28 (0.02)	1.16 (0.04)	0.006	1.04 (0.03)	1.07 (0.04)	0.432	1.45 (0.03)	1.52 (0.09)	0.445
Problematic gaming	0.44 (0.02)	0.59 (0.04)	0.001	0.69 (0.03)	0.65 (0.04)	0.428	0.25 (0.03)	0.31 (0.07)	0.441

**Table 2:** The prevalence (%, n) of on-site and online gambling during the past 12 months according to gender.

	Boys, % (n)	Girls, % (n)	Total, % (n)
No gambling	79% (1223)	97% (1518)	88% (2741)
On-site gambling only	7% (110)	2% (27)	4% (137)
Online gambling only	3% (54)	0% (3)	2% (57)
On-site and online gambling	11% (164)	1% (16)	6% (180)

**Table 4:** Factors associated with gambling **online** during the past 12 months among Finnish 15–16-year-old boys and girls in 2024, adjusted (AOR) odds ratios with 95% confidence levels (CL), *p* values for interactions of gender and independent variables from the full models.

	**Boys**		**Girls**		***p*** **(Interaction)**
	**AOR**	**95% CL**	**AOR**	**95% CL**	
Aiming at high school (ref = no)	1.51	1.01–2.28	0.39	0.13–1.21	0.0201
Truancy (ref = no)	1.26	0.86–1.86	3.92	1.17–13.08	0.0404
SES better (ref = same)	1.88	1.30–2.72	2.64	0.70–9.93	ns
SES worse (ref = same)	1.82	1.08–3.06	1.26	0.29–5.43	ns
High parental monitoring (ref = no)	0.61	0.41–0.91	0.92	0.29–2.98	ns
Hanging around with friends (ref = no)	1.28	1.07–1.53	0.78	0.40–1.48	ns
Risky sexual behavior (ref = no)	1.87	1.18–2.96	0.93	0.34–2.57	ns
Substance use 30 days/sum	1.39	1.24–1.56	1.59	1.04–2.43	ns
Problematic social media use 12 months/sum	1.12	0.91–1.38	0.89	0.56–1.41	ns
Problematic gaming 12 months/sum	0.92	0.75–1.13	0.41	0.13–1.31	ns

## Data Availability

The ESPAD trend data are archived at the Italian National Research Council (CNR), and the data can be used for research purposes (http://espad.org/databases, accessed on 5 March 2025).

## Appendix A. Pearson Correlations with *p*-Values of Original Continuous Independent Variables

	**Truancy**	**SES**	**Parental Monitoring**	**Hanging Around with Friends**	**Substance Use**	**Problematic Social Media Use**	**Problematic Gaming**
Truancy		0.081 <0.001	−0.103 <0.001	0.148 <0.001	0.368 <0.001	0.079 <0.001	−0.010 0.592
SES	0.081 <0.001		−0.011 0.539	−0.050 0.004	0.049 0.005	0.041 0.020	−0.005 0.788
Parental monitoring	−0.103 <0.001	−0.011 0.539		0.032 0.071	−0.106 <0.001	−0.005 0.789	0.013 0.462
Hanging around with friends	0.148 <0.001	−0.050 0.004	0.032 0.071		0.282 <0.001	0.027 0.113	−0.075 <0.001
Substance use	0.368 <0.001	0.049 0.005	−0.106 <0.001	0.282 <0.001		0.022 0.202	−0.018 0.292
Problematic social media use	0.079 <0.001	0.041 0.020	−0.005 0.789	0.027 0.113	0.022 0.202		0.337 <0.001
Problematic gaming	−0.010 0.592	−0.005 0.788	0.013 0.462	−0.075 <0.001	−0.018 0.292	0.337 <0.001	
